# Superinfection with Difficult-to-Treat Pathogens Significantly Reduces the Outcome of Periprosthetic Joint Infections

**DOI:** 10.3390/antibiotics10101145

**Published:** 2021-09-23

**Authors:** Ali Darwich, Franz-Joseph Dally, Khaled Abu Olba, Elisabeth Mohs, Sascha Gravius, Svetlana Hetjens, Elio Assaf, Mohamad Bdeir

**Affiliations:** 1 Department of Orthopaedic and Trauma Surgery, University Medical Centre Mannheim, Medical Faculty Mannheim, University of Heidelberg, Theodor-Kutzer-Ufer 1–3, 68167 Mannheim, Germany; franz.dally@umm.de (F.-J.D.); abuolba84@yahoo.de (K.A.O.); elisabeth.mohs@umm.de (E.M.); sascha.gravius@umm.de (S.G.); elio.assaf@umm.de (E.A.); mohamad.bdeir@umm.de (M.B.); 2 Institute of Medical Statistics and Biomathematics, University Medical Centre Mannheim, Medical Faculty Mannheim, University of Heidelberg, Theodor-Kutzer-Ufer 1–3, 68167 Mannheim, Germany; svetlana.hetjens@medma.uni-heidelberg.de

**Keywords:** outcome, difficult to treat pathogen, DTT, microorganism, superinfection, periprosthetic joint infection, PJI

## Abstract

Periprosthetic joint infection (PJI) is a serious complication after total joint arthroplasty. In the course of a PJI, superinfections with pathogens that do not match the primary infecting micro-organism may occur. To our knowledge, there are no published data on the outcome of such infections in the literature. The aim of this study was to assess the outcome of PJI with superinfections with a difficult-to-treat (DTT) pathogen. Data of 169 consecutive patients with PJI were retrospectively analyzed in this single-center study. Cases were categorized into: Group 1 including non-DTT-PJI without superinfection, Group 2 DTT-PJI without superinfection, Group 3 non-DTT-PJI with DTT superinfection, and Group 4 non-DTT-PJI with non-DTT superinfection. Group 3 comprised 24 patients and showed, after a mean follow-up of 13.5 ± 10.8 months, the worst outcome with infection resolution in 17.4% of cases (*p* = 0.0001), PJI-related mortality of 8.7% (*p* = 0.0001), mean revision rate of 6 ± 3.6 (*p <* 0.0001), and duration of antibiotic treatment of 71.2 ± 45.2 days (*p =* 0.0023). PJI caused initially by a non-DTT pathogen with a superinfection with a DTT pathogen is significantly associated with the worst outcome in comparison to non-DTT-PJI, PJI caused initially by a DTT pathogen, and to non-DTT-PJI with a non-DTT superinfection.

## 1. Introduction

Periprosthetic joint infections (PJI) are considered as one of the most serious complications after total joint arthroplasty (TJA). Occurring in 1% to 2% of primary total knee and hip arthroplasty cases [1,2,3,4,5] and in 5 to 15% of revision cases [5,6], it is considered as the most common cause of early failure after total knee arthroplasty (TKA) [7] and failure after total hip arthroplasty (THA) [8], representing almost 15% of all hip revision cases [9]. PJI have become a considerable challenge for infectiologists and orthopedic surgeons, creating a substantial economic burden and forcing prolonged periods of hospitalization and surgical revisions [4,10].

With a continuously aging population, the number of TJA performed per year all over the world is constantly increasing, consequently resulting in a growing number of concomitant PJI [11,12]. Despite the great interest and attention given to this issue, including improvements in the diagnosis and therapy algorithms, as well as the advances in preventive measures and perioperative preparations, such as laminar flow systems, perioperative systemic antibiotic prophylaxis, and decreased traffic in operating rooms [13,14,15], microbiological failure rates of up to 40% are reported in the recent literature after one- or two-stage revisions [16,17,18,19,20].

Alongside patient-related factors and misdiagnosis or delayed therapy [21], microbiological factors [22] and the entity of the infecting micro-organism [23,24,25] are considered as a few of the most important aspects affecting the outcome in PJI. For instance, the successful eradication of a Gram-negative PJI is far more difficult than that of a Gram-positive PJI, as per Hsieh et al. [26], who reported success rates of 27% versus 47% after prosthesis retention regimens.

In approximately 10 to 20% of cases, the infection is polymicrobial and, in 10 to 30% of cases, the cultures are negative [27,28]. This makes the treatment of the PJI and the choice of the most appropriate therapy regimen even more complicated.

Another key factor is biofilm formation and its crucial role in the pathogenesis and course of PJI. This important element led to the establishment of antimicrobial treatment regimens with biofilm-active antibiotics, such as fluoroquinolones in PJI involving Gram-negative biofilm-forming pathogens and rifampicin in PJI caused by staphylococci or similar Gram-positive biofilm-forming pathogens [29,30,31]. The high success rates of these antimicrobial strategies in comparison to non-biofilm-active antibiotics have been repeatedly confirmed in the literature [32,33]. Accordingly, micro-organisms for which no highly bioavailable biofilm-active antibiotic is available [5,34,35] are categorized as difficult-to-treat (DTT) [29,31,36] and pose an additional challenge in the treatment of PJI [5]. PJI caused by DTT pathogens (DTT PJI) are known to have a higher risk of clinical and microbiological failure, which, in turn, calls for an increased number of revisions; they require longer periods of antimicrobial treatment and respectively longer prosthesis-free phases [37,38,39].

In the course of treatment of PJI, where generally prolonged treatment regimens with multiple revisions are needed [40], superinfections with pathogens that do not match the primary infecting micro-organism may occur. Of special interest are superinfections with resistant pathogens, such as DTT pathogens that are not adequately covered by the initially administered antibiotic treatment. A classic example is PJI caused by Gram-positive pathogens and treated accordingly with antibiotics for Gram-positive organisms that show a superinfection with a resistant Gram-negative pathogen that is obviously not adequately covered by the initially administered antibiotic treatment.

Studies investigating the outcome of DTT PJI are sparse and involve mainly an individual pathogen [23,41,42]. DTT micro-organisms are either initially causing the PJI and are identified in the first surgical revision or appear at a later stage of the treatment and are identified in one of the subsequent necessary surgical revisions as a superinfection. To our best knowledge, there are no data analyzing the outcome after superinfection with a DTT pathogen in the course of the treatment of a PJI.

The aim of this study is to evaluate the prevalence of superinfections with a DTT pathogen in the course of a PJI and assess their effect on the outcome in comparison to non-DTT PJI, initial DTT infections, or superinfections with a non-DTT pathogen.

## 2. Results

A total of 169 patients with 169 hip or knee prostheses were included in this study. Group 1 consisted of 91 patients (53.8%). In 78 PJI patients, a DTT pathogen was involved: in 54 of them (69.2%), the DTT pathogen was detected in the first operation (Group 2) and, in 24 patients (30.8%), the DTT pathogen was considered as a superinfection and was detected at a later stage in the course of the PJI (Group 3). Group 4 comprised 16 patients (9.5%).

The recorded parameters did not significantly differ between groups. An overview of the characteristics of all patients, as well as of the formed groups, can be found in Table 1.

Regarding perioperative parameters, the mean interval duration between detection of the DTT pathogen causing the superinfection and the last revision without evidence of a DTT pathogen was 35.5 ± 47.2 days (7–179). A mean duration of 132.5 ± 195 days (8–901) was observed between the first revision and the surgical revision where the DTT pathogen causing the superinfection was detected.

The number of surgical revisions was also the highest in the group with DTT superinfections (Group 3), with a mean revision rate of 6 ± 3.6 revisions (2–14) (*p* < 0.0001). In this group, a mean of 2.4 ± 1.3 revisions were performed before detection of the DTT pathogen (*p* = 0.9917).

Concerning therapeutic concepts, the longest total duration of antibiotic treatment was also observed in the group of DTT superinfections (Group 3), with a mean of 71.2 ± 45.2 days (14–228) (of which 28.7 ± 7.5 days were orally administered) against 46.3 ± 29.5 days (4–217) (of which 28.3 ± 9.4 days were orally administered) in the group of non-DTT PJI with no superinfections (Group 1) and 61.1 ± 39.7 days (4–181) (of which 27.2 ± 12.1 days were orally administered) in the group of PJI with an initial detection of a non-DTT pathogen and no superinfection (Group 2) (*p* = 0.0023). An overview of all perioperative parameters can be found in Table 2.

The most detected DTT pathogens causing both initial DTT infections, as well as DTT superinfections were coagulase-negative staphylococci, followed by enterococci. Three cases of polymicrobial infection (two cases in Group 2 and one case in Group 3), each with a combination of a coagulase-negative staphylococcus and an enterococcus, were observed. All DTT superinfections were caused by new micro-organisms that were not detected in previous revisions. There were no superinfections with pathogen species matching the initial pathogen but with an upgraded resistance profile. 

A presentation of the DTT pathogen distribution can be found in Table 3.

The mean follow-up period was 13.5 ± 10.8 months (1–112). Seven patients (4%) lived abroad and were lost to follow-up. The number of patients free of infection (including all follow-up periods) was higher in Group 1 (67.9%) in comparison to all other groups (42.6% in Group 2, 17.4% in Group 3, and 43.8% in Group 4) (*p* = 0.0001). Similarly, the highest rates of therapy failure and PJI-related death were observed in Group 3, with 69.6% and 8.7% compared to 25% and 3.55% in Group 1 and 40.7% and 1.9% in Group 2, respectively (*p* = 0.0001). A summary of patient outcomes can be found in Table 4.

## 3. Discussion

PJI remains one of the most devastating complications after TJA [1,2,3,4,5]. With the continuously increasing number of TJA worldwide, as well as the surge of resistant organisms, the management of PJI continues to be a big challenge for orthopedic surgeons and infectiologists [23,25], and a huge financial burden on the healthcare system [43]. 

It is well established in the literature that the outcome of PJI is strongly affected by the type of infecting micro-organism and its resistance features [26,44,45]. In a recent animal model study, Masters et al. [46] showed the characteristics of implant-associated bone infection caused by *Staphylococcus aureus* and *Streptococcus agalactiae*. While osteotropic *Staphylococcus aureus* colonies were found in the osteocyte lacuno–canalicular network, *Streptococcus agalactiae* colonized blood vessels within cortical bone, revealing its more vasculotropic behavior. It is also documented that PJI involving resistant micro-organisms are associated with poor outcomes. Bradbury et al. [23], Tornero et al. [41], Deirmengian et al. [47], Chiu et al. [48], Walls et al. [42], and Tsumura et al. [49] investigated the outcome in patients with PJI secondary to methicillin-resistant Staphylococcus aureus and documented, according to the surgical treatment option, success rates varying from 16 to 100%. Kheir et al. [50] and Martinez-Pastor et al. [51] examined the outcome of PJI secondary to enterococci and to extended-spectrum beta-lactamase-producing Enterobacteriaceae and observed treatment success rates of 51.7% and 57.2%, respectively. Kuo et al. [52] examined treatment outcomes in patients with fungal PJI and reported success rates of 55.2% at 1 year and 40.5% at 5 years.

These studies assessed the outcome of PJI caused by resistant pathogens, but investigated just one particular type of micro-organism at a time. The outcome of PJI involving superinfections with DTT pathogens appearing at a later stage of the treatment and identified in one of the subsequent necessary surgical revisions has not yet been examined.

The present study reports considerably worse outcomes in patients with a superinfection with a DTT pathogen according to the criteria published by Laffer et al. [34]. Our study documents the influence of such a superinfection on both the infection resolution and therapy failure, as well as on death rates in comparison not only to non-DTT PJI, but also to PJI caused initially by a DTT pathogen and with PJI with a non-DTT superinfection. 

The comparison between PJI involving a DTT pathogen (Group 2 or 3) (where a DTT pathogen was detected at any point in the course of the infection) to non-DTT PJI (Group 1 or 4) (where no DTT pathogen was detected in the entire course of the infection) shows lower rates of infection resolution (42.6% and 17.4% versus 67.9% and 43.8%, respectively) and higher rates of therapy failure (40.7% and 69.6% versus 25% and 56.2%, respectively) (*p =* 0.0001). This result is expected, and it goes in line with the results already published by Wimmer et al. [53], who investigated the outcome of PJI involving DTT pathogens and also reported worse treatment success rates in the DTT group. Akgün et al. [37] also investigated PJI secondary to DTT pathogens and reported somewhat similar outcomes, but longer hospital stays, longer implant-free intervals, and longer antimicrobial treatment phases in the DTT group. These studies offer valuable data about DTT PJI; however, the timing of the first detection of the DTT pathogen in these studies was not recorded or considered.

In the current study, the further subcategorization of the DTT PJI showed that the rates of complete infection resolution, therapy failure, and PJI-related death were the worst in the group of patients with DTT superinfection (Group 3), where, initially, a non-DTT pathogen was the causative agent and the DTT pathogen was detected at a later stage in the course of the infection (17.4%, 69.9% and 8.7%, respectively) (*p =* 0.0001). These rates were shown to be even worse than those of patients (Group 2) with PJI caused by a DTT pathogen detected initially at the start in the first surgical revision (42.6%, 40.7%, and 1.9%) (*p =* 0.0001). Moreover, it was interesting that the rates in the group of patients with a non-DTT superinfection (Group 4) (43.8% and 56.2%, respectively) were also worse than those of the group of patients with an initial DTT (Group 2) (*p =* 0.0001). 

This means that the outcomes in PJI can be categorized in the following order (better to worse):

1. PJI caused initially by a non-DTT micro-organism where no pathogen switch is detected in the entire course of the infection (Group 1);
2. PJI caused initially by a DTT micro-organism from the start, detected in the first surgical revision where no pathogen switch is detected in the entire course of the infection (Group 2);
3. PJI caused initially by a non-DTT micro-organism with a superinfection or pathogen switch to another non-DTT pathogen in the course of the infection (Group 4);
4. PJI caused initially by a non-DTT micro-organism with a superinfection or pathogen switch to a DTT pathogen in the course of the infection (Group 3).

A possible explanation for this observation is the discrepancy in the number of performed surgical revisions per group [54]. Most revisions were performed in patients with a DTT superinfection (Group 3) (6 ± 3.6 revisions), followed by patients with a non-DTT superinfection (4.4 ± 2.9 revisions) (Group 4) (*p* < 0.0001). Multiple revisions are associated, among others, with multiple incision wounds with skin bridges, loss of soft tissue, and vascular insufficiency, which predicts higher complication rates [54] and may suggest that the local clinical findings may sometimes have a stronger effect on the outcome than the identity of the infecting micro-organism.

In fact, the effect of the systemic host status may be another explanation for the observed differences in outcome and is supported by two arguments: 

The first is that coagulase-negative staphylococci, which possess a very limited innate virulence [55], were the most abundant DTT found in the presenting cohort.

Secondly, the comparison of outcome between PJI with versus PJI without superinfection (Group 1 and 2 versus Group 3 and 4) shows worse outcomes in the first subgroup, independent from the DTT involvement (therapy failure: 62.5% versus 29.7%, *p =* 0.0002). This shows that the group of patients with superinfections involve the most probably primarily susceptible hosts that have fallen prey to opportunistic (super)infections that cannot be easily treated by surgical and antimicrobial interventions that were developed to cure infections from otherwise healthy tissues and hosts.

One of the limitations of this study is the low level of evidence, which is due to its retrospective and descriptive design. 

Some of the results in this study were obtained after the subcategorization of the PJI caused by DTT pathogen in several subgroups to highlight the effect of the time of appearance of the DTT pathogen in the course of the PJI on the outcome. A direct comparison of these results with those in the literature was not always possible due to the lack of such a subcategorization in the similar publications investigating PJI.

Another limitation is the rather small sample size in some of the analyzed subgroups, even though the study reports on a large number of patients with PJI and DTT PJI. A detailed subgroup analysis had to be clustered due to the small number of cases in the subgroups and the resulting marked limitation of statistical significance, so that a comparison could only be made between PJI with an initial DTT (Group 2) and non-DTT (Group 1), as well as DTT (Group 3) and non-DTT superinfection (Group 4), without taking into account the initial causative micro-organism. The group of patients with an initial DTT PJI and a DTT or non-DTT superinfection comprised a very small number of cases and was, for that reason, not considered in the comparisons. 

In addition, a further subdivision of the groups according to the affected joint and further reduction in the number of patients per group was not done, since the analyzed and established treatment concepts in the literature affecting the outcome are applicable for both joints. 

One more limitation is the lack of homogeneity in the measurement of patient outcomes in similar studies that also evaluated outcomes in PJI patients. This heterogeneity may have led to the observed discrepancy when comparing the results of the current study with those from the literature. For instance, Bradbury et al. [23] considered patients with a chronic antibiotic suppression a success. Kheir et al. [50] and Kuo et al. [52] adopted the Delphi consensus criteria [56] to define treatment success and did not consider CRP values to be an essential success criterium. On the other hand, Tornero et al. [41] and Chiu et al. [48] considered only prosthesis retention as a successful treatment.

In comparison with data in the literature, the definition of DTT was also heterogenous. In the current study, DTT pathogens were classified according to the criteria presented by Wimmer et al. [53]. However, several other studies [29,31,57] proposed other classifications that omitted some of the pathogens considered as DTT in the present study, or considered other resistance profiles as DTT, which limited the comparability of the results. 

Lastly, the surgical treatment of the included patients was performed by different surgeons that may have used different surgical techniques. Although the treatment follows the same well-defined internal algorithm, a small effect on the course of the infection cannot be excluded, which is considered as another limitation of the study.

## 4. Materials and Methods

### 4.1. Study Population

In this retrospective observational single-center study, all patients presenting or referred to our university hospital between 2015 and 2020 with a PJI were included. There were no exclusion criteria. The routine clinical data were collected and retrospectively analyzed.

### 4.2. Periprosthetic Joint Infection and DTT Pathogens

The management of all orthopedic-device-related infections in our university hospital is multidisciplinary and jointly directed by orthopedic surgeons and physicians from the institute of medical microbiology and hygiene, involving regular rounds to set and control all diagnostic and treatment aspects in order to provide the best treatment of PJI. 

PJI was diagnosed according to the new criteria proposed by Parvizi et al. [58] based on the criteria of the Musculoskeletal Infection Society (MSIS) [59].

Detected pathogens were scanned for the presence of DTT micro-organisms. In cases with a previously known causative pathogen and in emergency cases, a preoperative joint aspiration was not performed. Otherwise, the aspiration was routinely done. A portion of the collected aspirate was sent for cell count determination and cytological differentiation. The rest was sterilely inoculated immediately into blood culture vials [60].

Intraoperatively, a minimum of 4 double specimens were collected. Each double specimen consisted of 2 tissue samples from the same anatomical site. To allow matching of the results, one tissue sample was sent for microbiological culturing and the other for histopathological analysis. Cultures were considered negative if there was no growth of micro-organisms within 10 days [61]. Proof of a PJI in the histopathological examination of the intraoperative samples was based on the classification of Morawietz et al. [62]. The results and the detected pathogens were registered. Superinfections were also documented. 

As defined by Wimmer et al. [53], micro-organisms for which no highly bioavailable biofilm-active bactericidal antibiotic is available were categorized as DTT (Table 5). 

Rifampin and fluoroquinolones have excellent biofilm activity and offer a high bioavailability. *Staphylococcus* spp. resistant to these antibiotics were considered as DTT. 

Micro-organisms with a sensibility to only agents with low oral bioavailability, such as *Staphylococcus* spp. resistant to trimethoprim/sulfamethoxazole, doxycycline, and linezolid or *Enterococcus* spp. resistant to aminopenicillins, were classified as DTT, since alternative agents possess low oral bioavailability [53]. 

Micro-organisms that were only sensible to agents available for intravenous use, such as fluoroquinolone-resistant *Pseudomonas* spp. or yeasts resistant to azoles, were also categorized as DTT, since alternative agents are only available for intravenous use [53]. 

Slow-growing organisms, such as small colony variants (SCV), were considered as DTT, as they are characterized with an enhanced intracellular persistence [63] and gene expression involving formation of biofilms [64], allowing them to avoid antimicrobial activity [53].

Additional pathogens also considered as DTT but not detected in the patients included in the current study involved: fluoroquinolone-resistant *Acinetobacter* spp., since alternative antimicrobial agents are only available for intravenous use; *Cutibacterium* spp. due to the difficulty in their detection and eradication [65]; extended-spectrum β-lactamases (ESBL)-producing *Enterobacterales*, since this resistance is often combined with fluoroquinolone resistance, which make them only prone to agents available for intravenous use; and other slow-growing organisms, such as nutritionally variant streptococci.

After pathogen identification and consideration of sample sizes and clinical relevance, the following groups were formed:

1. Group 1: PJI with an initial detection of a non-DTT pathogen and no superinfection;
2. Group 2: PJI with an initial detection of a DTT pathogen and no superinfection;
3. Group 3: PJI with an initial detection of a non-DTT pathogen and a DTT superinfection item;
4. Group 4: PJI with an initial detection of a non-DTT pathogen and a non-DTT superinfection.

### 4.3. Recorded Parameters

Recorded patient parameters included age, sex, side, body mass index (BMI), preoperative comorbidity using American Society of Anesthesiologists (ASA) Physical Status Classification System [66], and preoperative anticoagulation. Surgical and antibiotic therapies that already started before admission were also documented.

Perioperative parameters included intraoperatively detected pathogen, results of the histopathologic examination of intraoperative tissue specimens, surgical treatment option, and duration of the antimicrobial treatment regime.

### 4.4. Surgical Treatment

Choice of the surgical treatment was met according to a standardized algorithm [40]. Treatment options involved debridement, antibiotics, and implant retention (DAIR) with exchange of its mobile parts, two-stage prosthesis exchange with a long 8-week interval between removal of the prosthesis and replantation, and multistage prosthesis exchange, where additional revisions and debridement, as well as spacer exchange (when applicable), were performed till the local findings were satisfactory for a replantation.

One-stage prosthesis exchange or two-stage prosthesis exchange with a short interval (2–4 weeks) were not performed.

Dual antibiotic-loaded bone cement was used for the cement spacers. Cement was prefabricated ready-made and not self-mixed. Antibiotic combination consisted routinely of 0.5 g gentamicin and 2 g vancomycin per 40 g PMMA, or, alternatively, of 1 g gentamicin and 1 g clindamycin per 40 g polymethylmethacrylate (PMMA). Selection of the cement was carried out according to the detected organism in previous surgeries [67]. 

Replantation was performed only when the local findings were satisfactory (healed surgical wound with no swelling, erythema, tenderness, or discharge from the incision site) and the laboratory results showed no signs of persistent infection (C-reactive protein (CRP) < 10 mg/L) [68]. In case a persistent infection was suspected, an additional surgical revision with debridement and specimen collection was performed. In cases with inserted spacers, the spacer was also exchanged.

### 4.5. Antimicrobial Treatment Regime

Intravenous antibiotics were regularly administered for the first 2 weeks, followed by oral antimicrobials for 4 weeks under close clinical and laboratory monitoring.

In cases of replantation, the surgery was planned only after satisfactory local findings and laboratory results following a 2-week antibiotic-free interval.

After replantation and according to the infecting pathogen, antibiotics were once again intravenously administered for 2 weeks, followed by oral biofilm-active antimicrobials for 4 weeks under close clinical and laboratory monitoring [40]. In cases with involvement of a DTT pathogen with a high resistance and a sensibility only to agents with low oral bioavailability or to agents only available for intravenous use, postoperative antibiotics were administered intravenously for 4 to 6 weeks.

### 4.6. Outcome

The end outcome was evaluated based on different clinical and microbiological factors, and was defined according to Laffer et al. [34] as one of the following:

- Free of infection:
  ○ Definitely free of infection: CRP < 10 mg/L, no clinical signs of infection, follow-up more than 2 years;
  ○ Probably free of infection: CRP < 10 mg/L, no clinical signs of infection, follow-up less than 2 years;
- Therapy failure:
  ○ Recurrence: Recurrence of the infection caused by the same pathogen or without evidence of pathogens;
  ○ New infection: Recurrence of the infection caused by a new pathogen;
  ○ Change of the surgical treatment regimen to resection arthroplasty, amputation, or chronic antibiotic suppression;
- Death due to sepsis;
- Death due to a non-PJI-related cause.

### 4.7. Statistical Analysis

All statistical calculations were performed using SAS software, release 9.4 (SAS Institute Inc., Cary, NC, USA). For quantitative variables, mean values and standard deviations were calculated. Categorical variables are presented as percentages, whereas continuous variables are presented as either mean ± standard deviation (SD) or median with interquartile range (IQR). In order to compare outcome groups regarding qualitative parameters, a chi-square or G test was used, where appropriate. For normally distributed data, a one-way analysis of variance (ANOVA) was performed to compare the mean values of different outcomes. Subgroup analysis was performed for the following groups: PJI with a non-DTT pathogen, PJI with an initial DTT pathogen, PJI with DTT superinfection in the course of the PJI, and PJI with non-DTT superinfection in the course of the PJI. A *p*-value of <0.05 was considered statistically significant.

### 4.8. Ethics Approval

This study was approved by the Ethics Committee of clinical research at our institution (Ethics Committee II, University Medical Centre Mannheim, Medical Faculty Mannheim, Heidelberg University, Theodor-Kutzer-Ufer 1–3, 68167, Mannheim, Approval 2021-814) and performed in accordance with the local ethical standards and the principles of the 1964 Helsinki Declaration and its later amendments.

## 5. Conclusions

This study confirms that PJI caused initially by a non-DTT pathogen with a superinfection of a DTT pathogen (Group 3) show the highest number of surgical revisions and are associated with failure rates of up to 69.6% in comparison to 56.2% in initially non-DTT PJI with a non-DTT superinfection, 40.7% in initially DTT PJI without superinfections (Group 2), and 25% in initially non-DTT PJI without superinfections (Group 1).

## Figures and Tables

**Table 1:** Patient data.

Patient Characteristics			Total (n = 169)	Group 1 (n = 91)	Group 2 (n = 54)	Group 3 (n = 24)	Group 4 (n = 16)	*p*-Value *
Age		Mean ± SD (range)	71.1 ± 13.1 (20–97)	70.6 ± 13.9 (20–93)	71.75 ± 10.9 (48–89)	71.1 ± 14.6 (35–97)	65.9 ± 10.9 (50–86)	0.6809
Sex	Female	n (%)	84 (49.7%)	47 (51.7%)	28 (51.9%)	9 (37.5%)	6 (37.5%)	0.5898
	Male		85 (50.3%)	44 (48.3%)	26 (48.1%)	15 (62.5%)	10 (62.5%)	
Side	Right	n (%)	81 (47.9%)	38 (41.8%)	26 (48.1%)	15 (62.5%)	5 (31.3%)	0.2663
	Left		88 (52.1%)	53 (58.2%)	28 (51.9%)	9 (37.5%)	11 (68.7%)	
Affected joint	Hip	n (%)	89 (52.5%)	39 (43.4%)	34 (63%)	15 (61.9%)	5 (30.8%)	0.8602
	Knee		80 (47.5%)	52 (56.6%)	20 (37%)	9 (38.1%)	11 (69.2%)	
BMI (Kg/m^2^)		Mean ± SD (range)	29.5 ± 6.5 (16.4–46.8)	28.8 ± 6.5 (17.6–46.8)	30.7 ± 6.3 (16.4–45.1)	29.5 ± 6.7 (18.5–42.3)	31.7 ± 7 (20–44.1)	0.3324
ASA Score		Median ± SD	3 ± 0.6	3 ± 0.6	3 ± 0.6	3 ± 0.8	2.5 ± 0.5	0.7147
Anti- coagulation	Yes	n (%)	67 (39.5%)	31 (34.1%)	24 (44.4%)	13 (54.2%)	2 (12.5%)	0.0743
	No		102 (60.5%)	60 (65.9%)	30 (55.6%)	11 (45.8%)	14 (87.5%)	

SD, standard deviation; BMI, body mass index; ASA, American Society of Anesthesiologists; * *p* < 0.05 indicates statistical significance.

**Table 2:** Perioperative parameters.

Parameters			Total (n = 169)	Group 1 (n = 91)	Group 2 (n = 54)	Group 3 (n = 24)	Group 4 (n = 16)	*p*-Value *
Treatment before admission	Antibiotic	n (%)	24 (14.2%)	13 (14.3%)	6 (11.1%)	5 (20.8%)	2 (12.5%)	0.8534
	Surgical		25 (14.8%)	11 (12.1%)	6 (11.1%)	5 (20.8%)	2 (12.5%)	
Antibiotic treatment	Total duration in days	Mean ± SD (range)	54.8 ± 36.7 (4–228)	46.3 ± 29.5 (4–217)	61.1 ± 39.7 (4–181)	71.2 ± 45.2 (14–228)	64.3 ± 47.3 (22–217)	0.0023
Surgical treatment	- Prosthesis retention	n (%)	66 (40.7%)	44 (52.4%)	15 (28.8%)	7 (30.2%)	4 (25%)	0.0080
	- Prosthesis exchange with cement spacer		75 (46.3%)	33 (39.3%)	31 (57.4%)	11 (45.8%)	12 (75%)	
	- Prosthesis exchange without cement spacer		21 (13%)	7 (8.3%)	8 (14.8%)	6 (24%)	0	
Replantation after prosthesis removal		n (%)	65/96 (67.7%)	29/40 (72.5%)	27/39 (69.2%)	9/17 (52.9%)	9/12 (75%)	0.6221
Number of revisions		Mean ± SD (range)	3.3 ± 3 (1–20)	2.3 ± 1.9 (1–14)	3.7 ± 3.3 (1–20)	6 ± 3.6 (2–14)	4.4 ± 2.9 (2–14)	<0.0001

SD, standard deviation; * *p* < 0.05 indicates statistical significance.

**Table 3:** Detected difficult-to-treat pathogens.

Pathogen	Total (n = 78)	Group 2 (n = 54)	Group 3 (n = 24)
Coagulase-negative staphylococci n (%)	47 (60.3%)	35 (64.8%)	12 (50%)
Enterococci n (%)	25 (32.1%)	15 (27.8%)	10 (41.7%)
Pseudomonas aeruginosa n (%)	5 (6.4%)	3 (5.6%)	2 (8.3%)
Candida albicans n (%)	4 (5.1%)	3 (5.6%)	1 (4.2%)
Polymicrobial * n (%)	3 (3.9%)	2 (3.8%)	1 (4.2%)

* 3 polymicrobial cases, each with a combination of a coagulase-negative staphylococcus and an enterococcus.

**Table 4:** Overview of patient outcomes.

Outcome		Total (n = 169)	Group 1 (n = 91)	Group 2 (n = 54)	Group 3 (n = 24)	Group 4 (n = 16)
Free of infection		84 (52.2%)	57 (67.9%)	23 (42.6%)	4 (17.4%)	7 (43.8%)
- Definitely free of infection		9 (5.6%)	7 (8.3%)	2 (3.7%)	0 (%)	2 (12.5%)
- Probably free of infection		75 (46.6%)	50 (59.5%)	21 (38.9%)	4 (17.4%)	5 (31.3%)
Therapy failure		59 (36.6%)	21 (25%)	22 (40.7%)	16 (69.6%)	9 (56.2%)
- Recurrence		20 (12.4%)	9 (10.7%)	8 (14.8%)	3 (13.1%)	4 (25%)
- New infection		19 (11.8%)	7 (8.3%)	8 (14.8%)	4 (17.4%)	3 (18.7%)
Change of the surgical treatment regimen		20 (12.4%)	5 (6%)	6 (11.1%)	9 (39.1%)	2 (12.5%)
Death		18 (11.2%)	6 (7.1%)	9 (16.7%)	3 (13%)	0
- Death due to sepsis		6 (3.7%)	3 (3.55%)	1 (1.9%)	2 (8.7%)	-
- Death due to a non-PJI-related cause		12 (7.5%)	3 (3.55%)	8 (14.8%)	1 (4.3%)	-

**Table 5:** Definition of “Difficult-to-treat” in the detected pathogens.

Pathogen	Resistance
*Staphylococcus* spp.	Rifampin Fluoroquinolones Oxacillin Trimethoprim/sulfamethoxazole Linezolid Doxycyclin
*Enterococcus* spp.	Aminopenicillin
*Pseudomonas* spp.	Fluoroquinolones
Yeast	Oral available azoles
Small colony variants (SCV)	

## Data Availability

The data presented in this study are available on request from the corresponding author.
